# Species delimitation and life stage association of *Propsilocerus* Kieffer, 1923 (Diptera, Chironomidae) using DNA barcodes

**DOI:** 10.3897/zookeys.957.54668

**Published:** 2020-10-12

**Authors:** Hai-Jun Yu, Xiao-Long Lin, Rui-Lei Zhang, Qian Wang, Xin-Hua Wang

**Affiliations:** 1 Key Laboratory of Aquatic-Ecology and Aquaculture of Tianjin, College of Fishery, Tianjin Agricultural University, Tianjin, 300384, China; 2 College of Life Sciences, Nankai University, Tianjin, 300071, China; 3 College of Fisheries and life Science, Shanghai Ocean University, Shanghai, 201306, China

**Keywords:** barcode gap, bioindicator, chironomid, COI, genetic distance, larval association, *Propsilocerus taihuensis*

## Abstract

The utility of COI DNA barcodes in species delimitation is explored as well as life stage associations of five closely related *Propsilocerus* species: *Propsilocerus akamusi* (Tokunaga, 1938), *Propsilocerus paradoxus* (Lundström, 1915), *Propsilocerus saetheri* Wang, Liu et Paasivirta, 2007, *Propsilocerus sinicus* Sæther et Wang, 1996, and *Propsilocerus taihuensis* (Wen, Zhou et Rong, 1994). Results revealed distinctly larger interspecific than intraspecific divergences and indicated a clear “barcode gap”. In total, 42 COI barcode sequences including 16 newly generated DNA barcodes were applied to seven Barcode Index Numbers (BINs). A neighbor-joining (NJ) tree comprises five well-separated clusters representing five morphospecies. Comments on how to distinguish the larvae of *P. akamusi* and *P. taihuensis* are provided.

## Introduction

**Figure 1.**
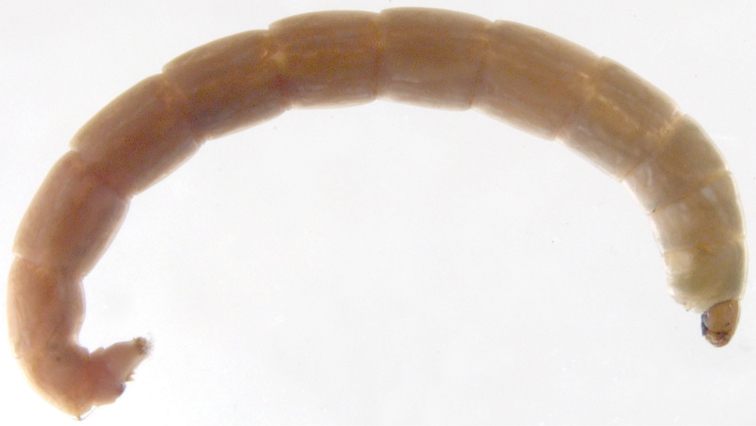
Larva of *Propsilocerus taihuensis* (Wen, Zhou & Rong, 1994).

The genus *Propsilocerus* Kieffer, 1923 (Fig. 1) was erected with the type species *Propsilocerus lacustris* Kieffer, 1923. At present, there are nine *Propsilocerus* species described in the Palaearctic and Oriental regions (Sæther and Wang 1996; Zelentsov 2000; Tang et al. 2004; Wang et al. 2007; Makarchenko and Makarchenko 2009) and one unnamed species from the Nearctic region (Cranston et al. 2011). Larvae of *Propsilocerus* usually inhabit eutrophic rivers and lakes. Because of their great densities and ability to adapt to different freshwater bodies, they are important food items for fishes and birds, and also bioindicators in monitoring of the freshwater ecosystem. However, the high morphological similarity between closely related species within *Propsilocerus* and intraspecific morphological variation have likely led to misidentifications, particularly in larvae. The morphological diagnosis (e.g., AR, LR_1_) of closely related morphospecies needs to be evaluated to verify the identity of each *Propsilocerus* species.

All four common species, *Propsilocerus akamusi* (Tokunaga, 1938), *Propsilocerus paradoxus* (Lundström, 1915), *Propsilocerus sinicus* Sæther et Wang, 1996, and *Propsilocerus taihuensis* (Wen, Zhou et Rong, 1994) are present in Yuqiao Reservoir, Jizhou Distinct, Tianjin, China during the spring and autumn. As a result, larvae of these four species usually have been misidentified as *Propsilocerus akamusi* by ecologists in China.

DNA barcodes (Hebert et al. 2003a, b) have proven successful in species delimitation and recognition of cryptic species diversity in chironomids (Anderson et al. 2013; Lin et al. 2015; Lin et al. 2018; Song et al. 2018). However, only one named (*P. akamusi*) and one unidentified species (*Propsilocerus* sp. JC-2015) have registered public DNA barcodes in the Barcode of Life Data systems (BOLD) (Ratnasingham and Hebert 2007). Thus, it is necessary to barcode more *Propsilocerus* species, which are common species in polluted rivers and lakes.

Here we explore the utility of DNA barcodes in species delimitation and in associating life stage in *Propsilocerus*. Registering new barcodes of *Propsilocerus* species will improve the reference library of Chironomidae (Ekrem et al. 2007) for DNA metabarcoding in biodiversity assessment in monitoring freshwater ecosystems.

## Materials and methods

In this study, 42 specimens of five *Propsilocerus* species (*P. akamusi*, *P. paradoxus*, *P. saetheri*, *P. sinicus*, and *P. taihuensis*) from China, Japan, Norway, and South Korea with COI barcodes were included. Twenty-six specimens with public COI barcodes were retrieved from BOLD and GenBank, and an additional 16 individuals of four *Propsilocerus* species were collected from the eutrophic lakes and reservoirs from Hebei Province, Shanghai and Tianjin, China, using D-nets, sweep nets, and light traps.

Larvae were preserved in 95% ethanol, adults in 85% ethanol, and stored at 4 °C in the dark before morphological and molecular studies. Photographs of all intact specimens were taken before dissection using a ZEISS camera mounted on a ZEISS stereomicroscope using the software AxioVision Rel. 4.8. at the College of Life Sciences, Nankai University, Tianjin, China. Digital photographs of slide specimens were taken at 300-dpi resolution using a Nikon Digital Sight DS-Fi1 camera mounted on a Nikon Eclipse 80i compound microscope.

Extraction of genomic DNA was done following the standard protocol of the Qiagen DNeasy Blood & Tissue Kit, except the volume of DNA template was 110 μl in the final step. Morphological terminology used in this work is according to Sæther (1980). The cleared exoskeleton of adults was mounted in Euparal on microscope slides together with the corresponding wings, legs, and antennae after DNA extraction. Voucher specimens from China were deposited in the College of Life Sciences, Nankai University, Tianjin, China.

DNA amplifications of COI barcode sequences with the universal primers LCO1490 and HCO2198 (Folmer et al. 1994) were carried out at the College of Fishery, Tianjin Agricultural University. Polymerase chain reaction (PCR) was set up using12.5 μl 2× Es Taq MasterMix (CoWin Biotech Co., Beijing, China), 0.625 μl of each primer, 2.5 μl template DNA, and 8.75 μl ddH_2_O to make a total of 25 μl for each sample. PCR was performed on a MasterCycler Gradient (Biometra GmbH, Göttingen, Germany), with an initial denaturation step of 95 °C for 4 min followed by 40 cycles at 94 °C for 45 s, 52 °C for 45 s, 72 °C for 1 min, and one final extension at 72 °C for 10 min. PCR products were electrophoresed in 1.5% agarose gel, purified and sequenced with ABI 3730 (BGI TechSolutions Co., Lit. Beijing, China).

Raw sequences were edited and assembled in SeqMan version 7.1.0 (in the LaserGene package, DNASTAR, Madison, USA), aligned using the Muscle algorithm (Edgar 2004), and checked for stop codons on the amino acids in MEGA version 7.0 (Kumar et al. 2016). Sequences were uploaded on BOLD with collateral information and images. A public dataset including all 42 specimens, “DNA barcodes of Propsilocerus [DS-PROPSIL]”, can be found in BOLD. The neighbor-joining (NJ) trees were constructed in MEGA using Kimura 2-Parameter (K2P) substitution model, 1000 bootstrap replicates and the “pairwise deletion” option for missing data. The pairwise distances of five *Propsilocerus* species were calculated using K2P model in MEGA. To detect the “barcode gap”, the aligned sequence dataset was subject to Automatic Barcode Gap Discovery (ABGD) (Puillandre et al. 2012) with the K2P model, following the default setting.

## Results

### DNA barcode analyses

**Figure 2.**
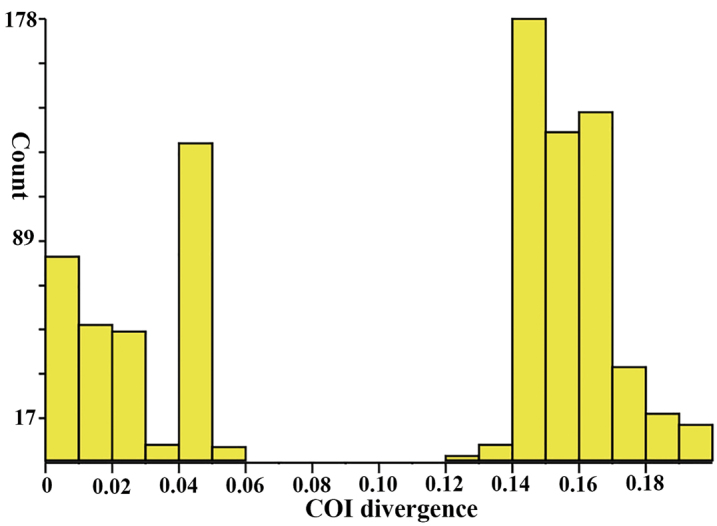
Histogram of pairwise K2P distances of 42 aligned sequences of five *Propsilocerus* morphospecies. The figure was a result of analysis with ABGD using the K2P model. The horizontal axis shows the pairwise K2P-distance, and the vertical axis shows the number of pairwise sequence comparisons.

In general, the data showed distinctly larger interspecific than intraspecific divergence, and there was a clear “barcode gap” in the pairwise K2P distances (Fig. 2). The minimum interspecific genetic distance between the closely related morphospecies *P. akamusi* and *P. taihuensis* is 13.4%. The maximum intraspecific distance of *P. akamusi* is 5.2%, 3.0% in *P. taihuensis*, 0.8% in *P. paradoxus*, and 0.5% in *P. saetheri* (*P. sinicus* is a singleton). Examining the present dataset in BOLD, 42 COI barcodes from five morphospecies of *Propsilocerus* were assigned into seven barcode index numbers (BINs). There are two BINs in each species *P. akamusi* (BOLD:ACB4994, BOLD:ACQ5058) and *P. taihuensis* (BOLD:ADX1391, BOLD:ADK5547), and a unique BIN in *P. paradoxus* (BOLD:ADX2356), *P. saetheri* (BOLD:AAM7072), and *P. sinicus* (BOLD:ADX6952).

**Figure 3.**
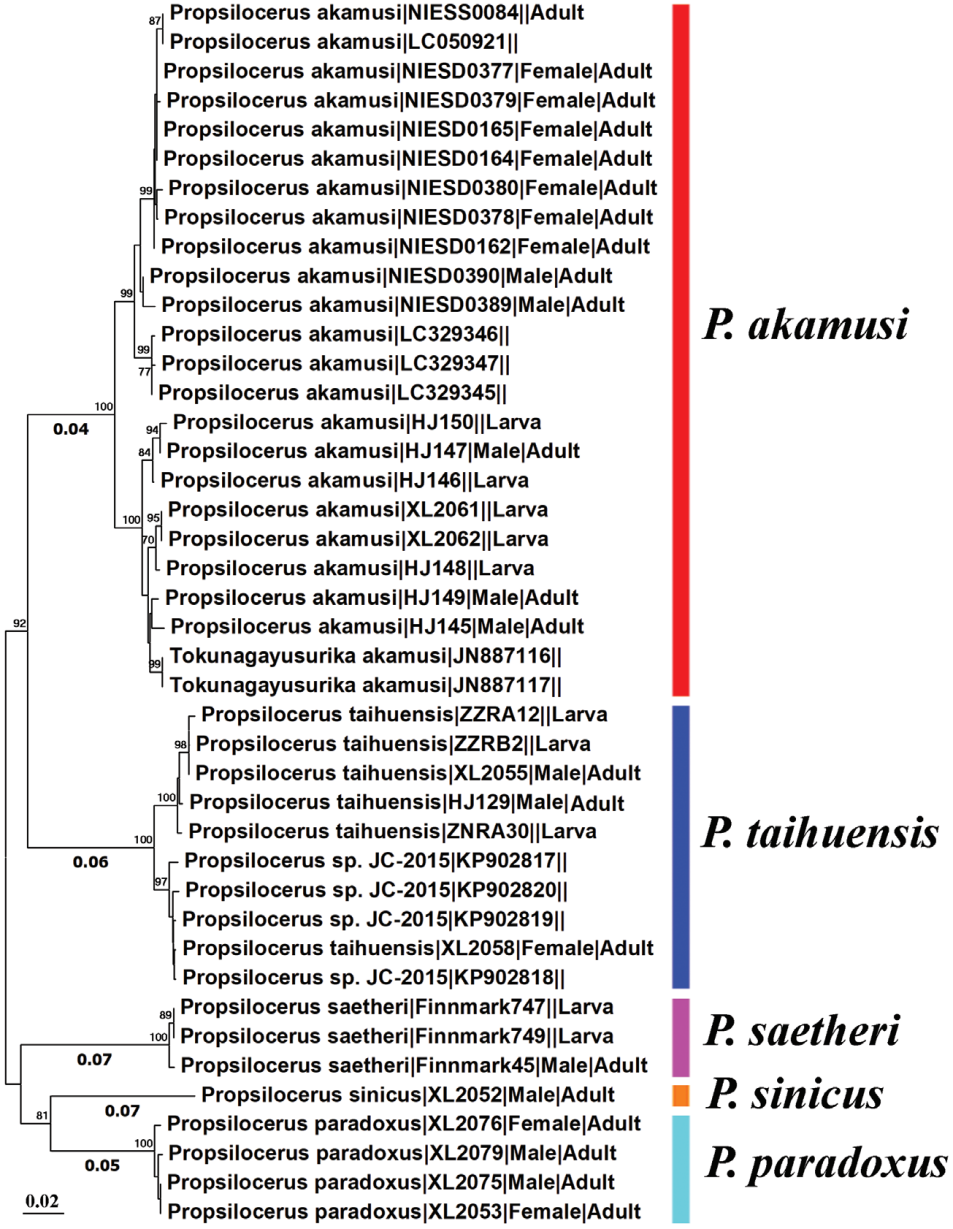
Neighbor-joining tree based on the 42 COI barcode sequences of *Propsilocerus*. Bootstrap support (1000 replicates) > 70% are labelled.

The neighbor-joining tree (Fig. 3) based on 42 COI barcodes of *Propsilocerus* species revealed five distinct genetic clusters, corresponding to five morphospecies. The unidentified species (*Propsilocerus* sp. JC-2015) grouped into *P. taihuensis* (Fig. 3). Larvae of *P. akamusi*, *P. saetheri*, and *P. taihuensis* can now be associated with adults based on DNA barcodes.

### Morphology

**Figure 4.**
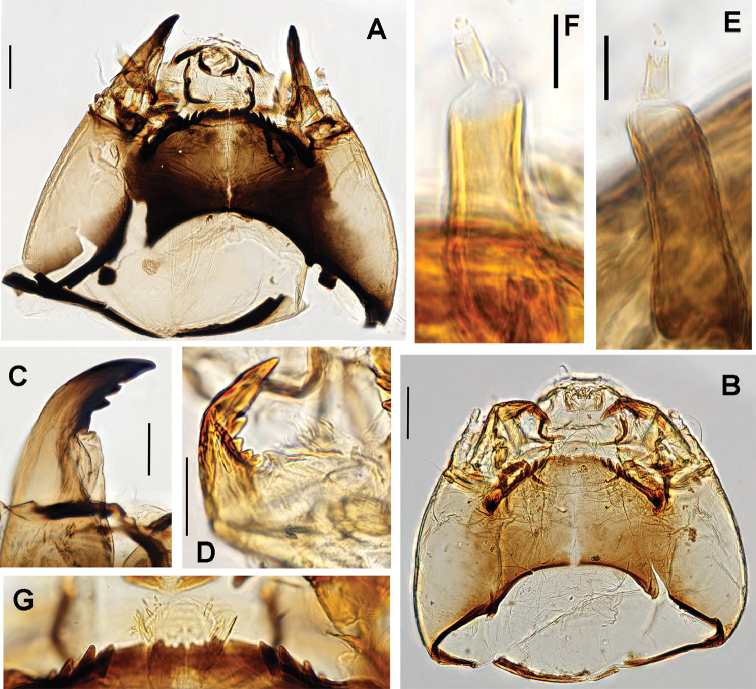
Head capsules of *Propsilocerus akamusi* (Tokunaga, 1938) and *Propsilocerus taihuensis* (Wen, Zhou & Rong, 1994) **A** head capsule of *P. akamusi*, ventral view **B** head capsule of *P. taihuensis*, ventral view **C** mandible of *P. akamusi***D** mandible of *P. taihuensis***E** antenna of *P. akamusi***F** antenna of *P. taihuensis***G** premento-hypopharyngeal complex of *P. taihuensis*. Scale bar: 100 µm (**A, B**), 50 µm (**C, D**), 25 µm (**E, F**).

Although it is feasible to distinguish species of *Propsilocerus* by referring to the works of Makarchenko and Makarchenko (2009), Sæther and Wang (1996), and Wang et al. (2007), misidentification of the larvae of *Propsilocerus* often occurs due to high morphological similarities. Currently, larvae of six *Propsilocerus* named species have been described. Tang et al. (2004) provided a key to the larvae of known species, and described the larvae of *P. taihuensis* based on the material from the type locality, Wuli Lake, Taihu Lake, Jiangsu Province, China. However, these larvae of *P. taihuensis* were not reared, and their identification could be uncertain. In this study, the larvae of *P. taihuensis* have been associated with adults using DNA barcodes. After reexamining the voucher and type specimens, we confirm that the description of the larvae of *P. taihuensis* by Tang et al. (2004) is correct, and *P. akamusi* can be separated from *P. taihuensis* by the relative lengths of the third and fourth antennal segments and the numbers of lateral teeth (Fig. 4) on the mentum (Tang et al. 2004). However, this is difficult in practice because larvae of *P. akamusi* and *P. taihuensis* both have dark head capsules, and 9–10 lateral teeth (often not easy to count) on the mentum, and short third and fourth antennal segments (Fig. 4). These two species can be more effectively distinguished by observing the premandible and mentum. In *P. akamusi*, the premandible is bifid, and the median portion of the mentum with one median notch and is subdivided into small teeth; whereas in *P. taihuensis*, the premandible is simple, and the median portion of the mentum has four teeth and one median notch.

It was also discovered that the undescribed Nearctic larva (Cranston et al. 2011) is closely related to *P. taihuensis* (Fig. 4B) in having a well-developed premento-hypopharyngeal complex (Fig. 4G) and the apical tooth longer and pointed, longer than the combined width of four teeth.

## Conclusions

Our study has revealed strong concordance between morphospecies and DNA barcodes of *Propsilocerus*. Distinct “barcode gaps” were discovered among *Propsilocerus* species. DNA barcodes have been used to associate different life stages, and the unidentified species (*Propsilocerus* sp. JC-2015) was confidently assigned to *P. taihuensis*. Comments on how to distinguish this species from congeners on the larvae of *P. taihuensis* are given.
